# Social Media as a New Vital Sign: Commentary

**DOI:** 10.2196/jmir.8563

**Published:** 2018-04-30

**Authors:** Sean D Young

**Affiliations:** ^1^ University of California Institute for Prediction Technology Department of Family Medicine University of California, Los Angeles Los Angeles, CA United States

**Keywords:** social media, big data, personal health records

## Abstract

Mobile technologies, such as wireless glucometers and mobile health apps, are increasingly being integrated into health and medical care. Because patients openly share real-time information about their health behaviors and outcomes on social media, social media data may also be used as a tool for monitoring patient care. This commentary describes how recent advances in computer science, psychology, and medicine enable social media data to become a new health “vital sign,” as well as actionable steps that public health officials, health systems, and clinics can take to integrate social data into both public and population health as well as into individual patient care. Barriers that first need to be addressed, including privacy concerns, legal and ethical responsibilities, and infrastructure support, are discussed.

## Introduction

Given the current discourse around social media, it might seem like “wall postings” have only existed since the turn of the millennium. However, people’s psychological desire to publicly share information has existed for thousands of years. For example, scribes, messengers, and other citizens of ancient Rome shared their thoughts on a literal wall—via graffiti—and elsewhere in Europe people exchanged pamphlets featuring diary entries, quotations, and personal information [1]. These snippets of daily life entertained and informed citizens, but they also provided a window into societal well-being. That is, they were a health indicator of sorts and served as one of the earliest forms of social media.

Today, unlike antiquity, health providers and researchers have access to an endless stream of personal data via the Internet and mobile devices. In one survey, 72% of Internet users searched for health information in the past year [2], and several studies have shown that social media can have a significant positive impact on health outcomes if it is properly incorporated into health care settings [3,4]. Given the tremendous amount of publicly accessible personal data (eg, >500 million tweets per day on Twitter), organizations and research teams are already starting to integrate social media services such as Twitter and Facebook into public health surveillance by tracking influenza, sexually transmitted diseases, and predicting crime more accurately than previous models [5-10].

Patients are willing to share highly personal information on social media, including thoughts, feelings, and behaviors that they typically would not disclose because of stigma or embarrassment [11,12]. Scientists have begun to search these data for important psychological clues about patient behaviors and outcomes, such as medication adherence and adverse reactions to medications [13,14]. By combining psychological insights with data science methods such as machine learning, it is possible to develop mathematical models that mine social media data to predict people’s health behaviors at a population and individual patient level. Researchers are debating the best methods to interpret and gain behavioral insights about social “big data” [15-17], but it is now feasible to monitor a host of lifestyle factors that affect a person’s mental health and physical well-being, such as stress level, physical activity, and sexual orientation.

## Real-Time Interventions

In a clinic or hospital, physiological vital signs such as blood pressure and heart rate are easy to monitor, but observant physicians also take note of psychological indicators such as sadness or anxiety. If physicians could detect when a patient is anxious, or why they are scared to take medications, for example, it might be possible to provide real-time interventions and improve care delivery.

Consider depression, the leading cause of disability worldwide. Depression costs the US health care system approximately US $200 billion annually and leads to negative health outcomes such as reduced medication adherence and suicide [18]. Although real-time monitoring of depression could save money and lives, it has been difficult to track outcomes outside of a clinic. This has changed with the advent of social media. Now, researchers can work together to analyze the words and images that people organically share on social media sites for clues about depression, drug abuse, or other psychiatric disorders. Algorithms can quickly identify patterns within millions of postings and use that information to predict people’s emotions and behaviors [19]. A check for suicidal ideation, for example, could be as simple as reviewing a list of keywords associated with suicide and depression [20,21]. However, despite the rapid progress in algorithm development over the years, there is still room for improvement. Algorithms implemented in a health care setting might need to apply Natural Language Processing techniques and require extensive additional research to optimize their accuracy.

While it will take time and research to develop automated algorithms capable of accurately identifying health risks within social data, we can already discuss more manual ways of implementing this approach, such as having trained staff available to review and discern whether social media posts that have been flagged by a machine for at-risk content actually do describe a patient’s valid health concerns and require attention. In fact, the process of having human domain experts identify and confirm health-related content, and teach this information to a machine, is a common approach used in developing new models for monitoring and prediction [17].

With the right infrastructure and clear and informed consent from patients, it might be possible for people’s real-time, organic social media data to be used as a source of information about their health and vitals. New health care tools could be developed and used based on this information to help alert health systems and their staff about potential or ongoing patient risks. How can this be done? Health systems could ask patients for permission to review their social media history, along with consent to monitor online activity when admitted to a hospital or medical facility. If a patient were to repeatedly express patterns of concern, such as talking about “pain,” “being depressed,” or “suicide,” machines that mine this information could flag it for a health system staff member to review and intervene if deemed necessary.

Similar approaches to mine social media data can also be applied at the public health population level to help researchers and public health officials identify epidemics. This has already been the case for HIV [12,22], influenza [23], and cardiovascular disease [24]. With some refinement, social media surveillance strategies may be able to inform early warning systems and facilitate communication between doctors and public health authorities. Once a patient, population group, or geographic region of concern is identified, providers and health officials would need to follow a strict protocol to maintain privacy and confidentiality.

## Roadblocks

Once equipped with these tools, providers and public health officials could have real-time access to monitor high-risk patients. However, there are a number of implementation questions that first need to be resolved. One question is whether providers and public health officials would be responsible for acting on social media data, as when providers identify a patient with high blood pressure. Because social media postings occur continuously 24 hours a day, 7 days a week, inside and outside of hospitals and clinics, this could pose a tremendous legal and economic burden on health care facilities to monitor and act on this information. For example, ethicists, lawyers, and health system management would need to discuss whether, how, and when providers should monitor and intervene. For example, if a patient posts on the social media platform of their cardiologist about a heart problem, would the cardiologist be responsible for following up? If the patient did not discuss cardiology but instead described mental health problems, an area outside of cardiology, would the responsibilities of the cardiology team change? These are some of the many unexplored questions that would need to be discussed. Before implementation of monitoring patient social data, there should be clear and ethical protocols in place to manage these situations such as having a mental health professional on-call to deal with potential crises [25].

Given that there are a limited number of studies showing a causal link between social media posts and actual health outcomes due to the novelty of this area, we suggest that it is too early to assign responsibility for monitoring social media as a health outcome. Instead, at this stage it might be used as an additional tool, instead of a standalone one, for remote monitoring of health and additional insights about patients.

Using social media to monitor and predict health risks also raises serious privacy concerns. For example, are patients willing to have their data mined to monitor their health information? Although studies on ethics suggest that people find social media to be an acceptable tool for use within public health and medicine [26,27], this is a constantly changing area. It is unclear whether people who share their data today would be willing to have their data used in the future. A recent systematic review shows that there are mixed views on the ethics of social media research, with privacy concerns being the biggest hurdle. Despite the public nature of social media, many people expressed more positive views about social media research when studies required informed consent from participants [28]. Another concern is that some people using social media may have their profile set to public and be unaware of the privacy risks involved or have forgotten that their profiles are public. It is unclear whether these or other individuals are comfortable sharing personal health information with a health care provider. Hospitals and health care systems that implement a method of following patients on social media should therefore have consent forms that explicitly remind patients when and if they would be monitored and describe the currently known risks and benefits of this approach.

Another issue to consider is the data storage and management system. Social data could be managed within an electronic health records system or managed in a separate system, such as by clinics or health systems. Pilot testing is needed to address these questions before rolling out implementation at the larger level of electronic health records or health systems. For example, feasibility studies with small clinics will help uncover potential risks that need to be addressed before widespread implementation

Finally, there is also concern around the validity of the data. People can provide inaccurate information about their health and behaviors on social media. For instance, during the 2011-2012 flu season, flu-related Twitter models suggested that it was a typical season. However, after World Health Organization data showed that flu prevalence peaked 3 months later than a typical flu season, it was discovered that the Twitter flu model had incorrectly characterized tweets as being related or unrelated to flu. More research is therefore needed to ensure the accuracy of monitoring illnesses via social media, especially during atypical seasons [29].

## The Future of Social Media and Health Care

Finally, before implementing social media as a tool for monitoring data, we need to address whether providers and health systems might not want or know how to handle this additional information about their patients. With the increasing amount of data available, it can be overwhelming to a health system to think about integrating an unfiltered, 24-hour source of information into health services and even more daunting to think about the possible risks that could occur from being one of the first to implement an approach like this. This paper is meant to initiate a discussion of the risks and benefits of this approach in order to determine whether and how social media might be integrated into health monitoring to improve public health and medical care. Although widespread implementation of social media monitoring in health care may be a number of years away, there are already case studies that use social media and other forms of big data as a tool for monitoring in other fields.

Tools to analyze social data are already successfully being used in fields like consumer behavior, education, and crime prediction [30], and there are a growing number of platforms that will soon be ready for wide-scale use by medical professionals. For example, our team at the Institute for Prediction Technology has been developing a visualization tool called Cloudberry-HIV Map [31]. We are building visualization tools like this to mine social media data and present this information to public health experts in an easy-to-understand manner so they can access “big social data” and use it to assist with the interventions and resource allocations. We have been working on applying these types of models to address HIV, natural disasters, drug addiction prevention, and suicide prevention. To date, the most extensive use of machine learning for public health in the private sector is IBM’s Watson Health, a computer that mines patient data to aid in diagnosis and treatment. In 2011, researchers started feeding the supercomputer millions of academic research papers and programmed it to understand standard treatment protocols for cancer. In one case, IBM claims that Watson Health proved better than doctors at diagnosing a rare form of leukemia [32]. Watson Health has gone through extensive testing and refinement, but it has been effective at analyzing patient medical records, identifying cancer treatment options, and providing supporting evidence for its treatment recommendations. Developing a supercomputer that can be used in a broader fashion with social media (eg, to identify early forms of mental illness) is a next step. At a minimum, it is currently possible to train computers to comb social media accounts and identify patients at risk for diseases such as depression [33]. Working with a multiplatform, rapidly evolving technology may indeed be intimidating, but research in this area suggests it could have tremendous benefits to patients and to broader public health. For this to happen, health providers, patients, and computer scientists will need to start a dialogue. The Health Insurance Portability and Accountability Act and protected health information guidelines are an important topic of discussion. Particular focus needs to be applied to whether public social media posts should be considered protected health information. In addition, infrastructure to support these tools and patient willingness to participate are a few other considerations. In some sectors this dialogue has already begun, but to make progress we need to shift our mindset on the role of social networking technologies. As health researchers and providers, being open to thinking of social media in this manner—as a potential new “vital sign”—is a wise and worthwhile change in perspective.
